# Assessment of Dysphagia Management Competence among Healthcare Providers: A Scoping Review

**DOI:** 10.1007/s00455-025-10835-1

**Published:** 2025-05-28

**Authors:** R. Jordan Hazelwood, Garnet F. Robinson, George W. Wolford, Rebecca F. Smith

**Affiliations:** 1 Department of Rehabilitation Sciences, Beaver College of Health Sciences, Appalachian State University, 1179 State Farm Road, LLHS 494, Boone, NC 28608 USA; 2 Pediatric Therapy Specialists, Prisma Health-Midlands, 14 Richland Medical Park Drive, Suite 100, Columbia, SC 29203 USA; 3 Department of Rehabilitation Sciences, Appalachian State University, Beaver College of Health Sciences, 1179 State Farm Road, Boone, NC 28608 USA; 4 School of Communication, Roxelyn and Richard Pepper Department of Communication Sciences and Disorders, Northwestern University, 2240 Campus Drive, Evanston, IL 60208 USA

**Keywords:** Dysphagia, Competence, Assessment, Training, Healthcare provider

## Abstract

Functional swallowing is imperative to sustain life and maintain health, necessitating healthcare providers’ competence in managing swallowing disorders. This scoping review aims to identify and compare how competence in dysphagia management is assessed among healthcare providers. Our search identified 11 final records, demonstrating the limited existing literature. Overall, no specific standardized protocol currently exists for the assessment of dysphagia competence across healthcare disciplines. Therefore, developing a standardized metric to assess competence in dysphagia management among healthcare providers would not only improve training in dysphagia management by creating a consistent standard for healthcare providers’ competence, but also promote equitable and effective care delivery across diverse healthcare settings, improving outcomes for individuals with swallowing disorders on a global scale.

## Introduction

Dysphagia can have detrimental health and social consequences such as malnutrition, pneumonia, dehydration, decreased quality of life, and even death [1–8]. The substantial financial and caregiver burdens associated with dysphagia can also lengthen hospital stays and increase caregiver responsibilities [9–14]. These burdens manifest as higher health insurance costs for patients with more severe dysphagia [15], negative mealtime experiences [16], and caregiver distress [17]. Due to these significant impacts, healthcare providers are expected to comprehensively assess swallowing disorders, support the accurate diagnosis of dysphagia, and provide individualized patient care [18–20]. Dysphagia management requires complex equipment and specialized clinical skills and assumes awareness of collaborative practices[21–24]. Therefore, well-trained professionals are needed for optimal dysphagia management.

Interdisciplinary care for dysphagia management is considered the best practice to reduce complications, resulting in better health outcomes and decreased costs [25–27]. Interdisciplinary care occurs when a team of professionals with differing perspectives and management approaches provide holistic care to patients with dysphagia and their caregivers [28]. Despite the benefits of this model, the composition and implementation of interdisciplinary care teams can vary significantly based on facility type and geographic location [29]. Internationally, challenges such as differences in training, affordability, and access to care further complicate establishing and conceptualizing such teams [30]. Additionally, professionals managing dysphagia have increasingly assumed overlapping roles without clear training standards for given competencies [31–33]. This has led to pervasive inadequacies in provider training and competence, compounding substantial disparities in dysphagia education across various healthcare settings [32, 34–38]. These inadequacies pose a serious concern as they directly impact the quality of care delivered to patients with dysphagia [34, 39, 40]. To combat this, all providers, regardless of profession, should achieve minimal competence at certain training levels for dysphagia management [32, 35, 41].

A standardized, competence-based assessment could mitigate these issues by promoting uniformity in practice and improving clarity in documentation, which would foster better collaboration across clinical teams [30, 42, 43]. A well-designed, competence-based assessment determines whether an established performance or competence standard has been met for the practitioner [44]. These metrics are specifically designed to evaluate the competence of the healthcare professional, focusing on their knowledge and skills rather than assessing the eating, drinking, and swallowing of the patients being evaluated. The *Assessment of Clinical Skills/Competence/Performance* framework indicates four domains of competence to be included in a comprehensive assessment: knowledge, competence, performance, and action [45]. High-quality, comprehensive competence assessments should evaluate healthcare providers across a broad range of domains using a mix of self-reported outcomes, global ratings of competence, and skill-based checklists of critical dysphagia management techniques [36, 43, 46]. For instance, practitioners’ competence should be assessed on both subjective clinical skills components (e.g. assessing the quality of observations made during a clinical swallow evaluation) and instrumental skills (e.g. evaluating the accuracy of anatomical measurements and physiologic impressions during an instrumental exam). These competence assessments should be standardized in their administration and extend beyond knowledge-based evaluation to ensure a thorough appraisal of skill-based clinical competencies [44, 45]. Given this, a measurable set of competencies and minimal levels of competence should be established to support professional development across various levels of training [32, 44, 46]. Therefore, this scoping review aims to identify and compare how competence in dysphagia management is assessed among healthcare providers.

## Methods

### Scoping Review

Scoping reviews map evidence of a topic and identify the main concepts, theories, sources, and knowledge gaps [47]. Given that our research question focuses on discovering the breadth of information about competence in dysphagia management across multiple healthcare disciplines, this study methodology is optimal for exploring current literature. This scoping review was performed according to the Preferred Reporting Items for Systematic Reviews and Meta-Analyses Extension for Scoping Reviews (PRISMA-ScR): Checklist and Explanation to describe ways competence in dysphagia management is being assessed among healthcare providers [47].

### Database Search

The research question that guided our search was, “How is competence in dysphagia management assessed among healthcare providers?” To best answer our question, we included databases that would return a wide array of records, such as published manuscripts, conference proceedings, book chapters, and published dissertations in healthcare, to broaden the search. Our electronic search strategies were designed and tailored for each database with the support of a university librarian. Our search was conducted on February 4, 2023, in the following databases from the earliest available data to the current year: PubMed (1982–2023), CINAHL (2003–20232), Digital Commons Network (DCN), Medicine and Health Sciences (1901–2023), and WorldCat (1928–2023). We selected these databases in consultation with our university librarian to ensure a comprehensive approach, capturing resources across multiple disciplines, including grey literature. Additionally, reference lists of included articles were reviewed for other potential records following the full-text review. The quality of individual records was not appraised for this review.

All databases were searched using keywords selected according to the controlled descriptors for Medical Subject Headings (MeSH) and Exact Subject Headings (MH). Due to the nuanced distinction between the terms competence and competency, Boolean truncation was used, if available. See **Appendix A**. While competence refers to the overall ability, skill, or capacity to perform a task effectively, competency refers to specific, measurable skills or attributes required for successful task execution [43, 45]. Including both term possibilities in the search allowed us to capture a broad range of studies that examined the assessment of dysphagia management across various healthcare professions, regardless of whether the focus was on general professional ability (competence) or specific skill sets (competencies).

### Record Selection

All records were stored using Zotero reference management software [48]. Two reviewers (CAM and GFR) independently assessed all included titles and abstracts and determined the record’s eligibility as “yes” (included) or “no” (excluded). This process was completed using Covidence, a systematic review management software [49]. When the reviewers disagreed about the eligibility of any record, conflicts were resolved with a third reviewer (RJH) as the tiebreaker.

### Study Eligibility

Three reviewers completed the title/abstract screening, the full-text review, and the hand search [50]. Training on agreement was conducted as a group of three, requiring consensus agreement on five studies randomly selected from the search before beginning all phases.

Following our literature search, we screened the titles and abstracts of all the non-duplicated studies resulting from our searches. We applied the inclusion criteria and exclusion criteria. Following the title and abstract screening, we read the full article using the inclusion and exclusion criteria. See Table 1. We specifically included keyword variants to capture our two main ideas: dysphagia and competency. Our decision to restrict resources to English-only text was practical. Additionally, we wanted to focus on curated resources published for a professional clinical audience and not textbooks for trainees or the general public.

**Table 1:** Exclusion and inclusion criteria

Inclusion criteria	Exclusion criteria
Title/Abstract Screen	
Keywords related to dysphagia (e.g. deglutiti*, swallow*, dysphag*, etc.)	Record is not published in English
Keywords in any form of competen*	Record is not an article, periodical, dissertation, or thesis (i.e. book)
Full-Text Review	
Describes a protocol used to measure the concept of competence in dysphagia	Record is not an article, periodical, dissertation, or thesis (i.e. book)
Used an aforementioned protocol to measure the concept of competence in dysphagia management of a healthcare provider	Unable to obtain the full text after attempting all sources available

Upon completing our full-text review, all three reviewers independently completed a hand search of all references from all included studies using the same title and abstract screening procedures detailed in Table 3. From there, all three reviewers met and applied the full-text criteria to qualifying studies, yielding one additional study.

### Data Extraction

One reviewer (GFR) completed data extraction for all records that met all inclusion criteria. Data extraction included the following factors: author, year, country of publication, type of record, type of data, type of study, author discipline, assessment protocol, assessment protocol scoring, assessment protocol focus, skills assessed, population of interest, sample size, setting, and database. See Table 2.

**Table 2:** Operational definitions of data extraction factors

Data extraction factor	Operational definition
Author	Record authors
Publication Year	Record year of print
Publication Country	Corresponding author’s country address
Record Type	Record publication type
Data Type	Record data type (qualitative, quantitative, or mixed)
Study Type	Record research design
Author Discipline	Discipline of the lead author
Assessment Protocol Name	Name of protocol used to assess competence in dysphagia management
Assessment Protocol Scoring	Scoring procedures for assessment protocol
Assessment Protocol Focus	Area of dysphagia assessed within assessment protocol
Skills Assessed	Skills assessed within the assessment protocol
Population of Interest	Discipline(s) of participants and/or students
Sample Size	Number of participants
Participant Setting	Setting of participants
Database	Database from which the record was retrieved

## Results

**Fig. 1:**
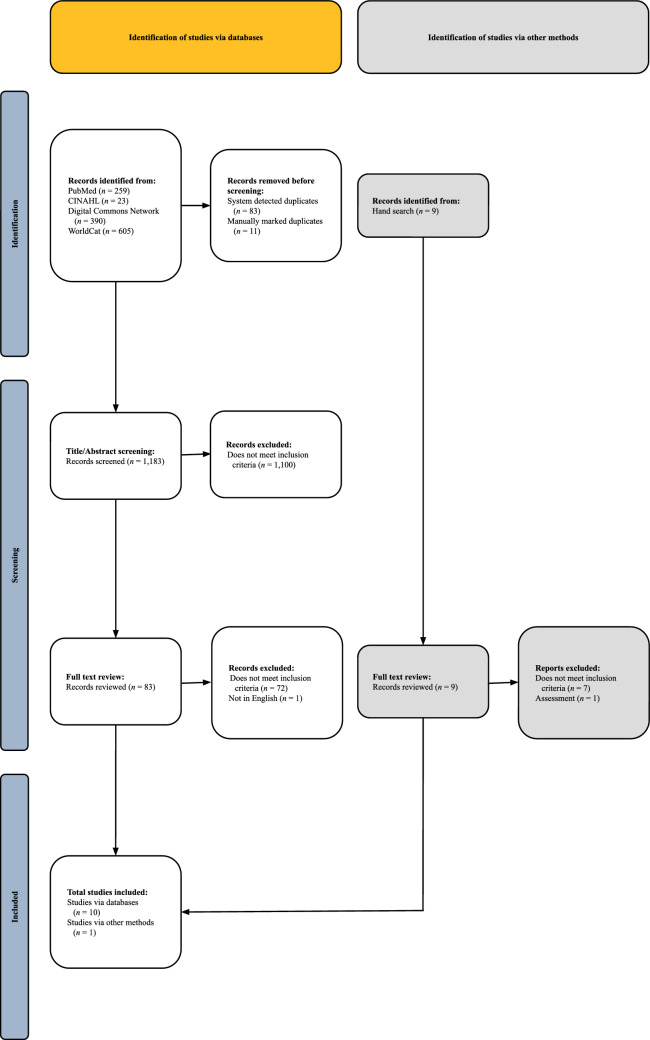
PRISMA-ScR flow chart

Results from our database searches were imported into Covidence (*n* = 1,277): 259 from PubMed, 23 from CINAHL, 390 from DCN, and 605 from WorldCat. Ninety-four total duplicates were removed. Eighty-three were system-detected duplicates, while 11 were manually marked as duplicates. After removing the duplicates, 1,183 abstracts and titles were screened for inclusion. Interrater reliability was high at this step (*k* = 0.865). After screening titles and abstracts, 83 full-text records were reviewed for inclusion. Interrater reliability was relatively low at this step (*k* = 0.423) due to the lack of a universal definition of competence. Reviewers displayed more difficulty with the agreement during the full-text review due to the word “competence” being written as “competency”, “skills checklist”, and “knowledge, attitude, and skills” [36, 51]. To ensure we included the correct articles, the reviewers met to discuss all full-text discrepancies noted in Covidence, confirming agreements by discussing the rationale behind differing judgments, ensuring consistency, and achieving full consensus in applying the criteria. Of the 83 full-text records, one met exclusion criteria (not in English), and 72 did not meet inclusion criteria. Therefore, we included ten records at the full-text review stage. The hand search yielded one additional record following the full-text review. In total, 11 records were included in the final corpus for data extraction. See Fig. 1. Data extraction details were gathered according to our operational definitions. See Tables 2 and 3.

**Table 3:** Summary of resource details, competency assessment information, and context

Author	Year	Country	Record type	Data type	Study design	Author discipline	Assessment protocol name	Assessment protocol scoring	Assessment protocol focus	Skills assessed	Population of interest	Sample size	Participant setting	Database [52]
Arsenault & Atwood [52]	1991	USA	JA	N/A	NAS	SLP	MGH protocol for developing independence in the diagnosis and treatment of dysphagia with practicing clinicians (on-the-job- training)	• 4 Phases: 1) Observation and acquisition of basic skills and knowledge (general knowledge of competency) 2) Directed experience (demonstrates competency on 10 consecutive patients with 90% agreement) 3) Indirect supervision (demonstrated competency on 10 consecutive patients with 100% agreement) 4) Intermittent supervisory monitoring (demonstrated competency on 10 consecutive patients with 100% agreement)	• Use/ knowledge of instrumenta- • tion • Theoretical knowledge of dysphagia • Competency in treatment • Competency of CSE	• History collection • Treatment programs • Information dissemination • Interdisciplinary communication • Theoretical knowledge • CSE • Instrumental and non-instrumental assessment procedures • Appropriate methods of documentation • Department and hospital policies and procedures • Appropriate referrals • Data interpretation • Prevention methods • Patient education and counseling • Total management of dysphagic caseload	• SLP •SLPSt	N/A	Medical SLP setting	World Cat
Browner & Bessier [32]	2004	USA	JA	QN	XS	NUR	Competency fair	• Not enough information was provided	Competency in treatment	• Various diet consistencies • Restorative feeding program • Caregiver education • Feeding/ swallowing safety	All rehab disciplines	95	Rehab setting	PubMed
Freeland et al. [56]	2015	USA	JA	QN	XS	SLP	Medical mannequin training and didactic training and assessment	• Scored by researchers using a pass/fail system • Accurate administration (100%) was defined as correctly performing the six skills • The process was repeated until every trainee reached a score of 100% • Scored immediately after training, at 2 weeks, and at 4 weeks with a medical mannequin • Scored at 6 weeks post-training with standardized patients (SLPs)	Competency with CSE	• Administration and interpretation of swallow screening • Administration and interpretation of swallow screening • Positioning of patient • Administration of swallow screening items	NUR	32	VA Medical Center	PubMed & World Cat
Hazelwood et al. [37]	2022	USA	JA	QN	XS	SLP	Modified DCVT	• 5-point ordinal scale with responses 0–4 (absent, dependent, emerging, adequate, excellent) • Scored by graduate students (self-perceived)	• Use/knowledge of instrumentation • Competency of CSE • Competency in treatment	• CSE and dysphagia treatment– general skills • CSE and dysphagia treatment– direct patient care • VFSS • FEES	SLPSt	72	Academic	Digital Commons Network
Hoepner & Hemmerich [53]	2020	USA	JA	QN	XS	SLP	Video and live competency performance assessment	• Scored by authors 1, 2, and another colleague • No detailed information was provided	• Competency of CSE	• Administration and interpretation of OME & CSE • Observations during OME & CSE • Appropriate documentation and referrals	SLPSt	43	Academic	PubMed & CINAHL
Kennedy et al. [54]	2019	UK	JA	QN QL	MIX	PSYSt	VAS tool	• Respondent places a mark at a point along a line with endpoints of 1 (little knowledge) to 10 (very good knowledge or skills) • The mark on the VAS was measured from the endpoint (1) and calculated arithmetically as a percentage of the total measurement between 1 and 10 given pre- and post-education on the subject	Not enough information was provided	Six domains of aging & health curriculum: 1) History 2) Exam 3) Drug Use 4) Comorbidities 5) Nutrition 6) Swallow	MDSt	197 pre-assess- ment; 201 post- assess- ment	Academic	PubMed
Luo et al. [19]	2022	China	JA	QN	XS	NUR	Questionnaire	• 4 phases of assessment: 1) qualitative data 2) multiple choice questions- incorrect answers scored as 0 and correct answers scored as 5 with a total range from 0 to 100 3) 5-point Likert scale with responses 1–5 for attitudes (completely disagree, disagree, neither agree nor disagree, agree, or completely agree) 4) 5-point Likert scale with responses 1–5 for practices (never, sometimes, half of the time, most of the time, or always) • The higher the score, the stronger the ability of the geriatric nurse regarding dysphagia care	• Theoretical knowledge of dysphagia • Competency in treatment • Competency of CSE	• Theoretical knowledge of dysphagia • Attitudes towards dysphagia care • Management of dysphagia in elderly patients • Risk factors of dysphagia • Clinical manifestations • Assessment methods • Intervention measures	NUR	782	Hospital	PubMed
McAllister [42]	2005	AUS	Thesis	QN QL	MIX	SLP	Competency-based assessment VAS	• VAS from novice to intermediate to entry-level • Left-anchored option for "not observed” and right-anchored option for "above entry level” • Mark made at mid-placement assessment and at end placement assessment • Clinical Educator and student complete VAS	• Competency of CSE • Competency in treatment	• Administration and interpretation of swallow assessment • Intervention plan • Maintenance and delivery of service • Professional, patient, caregiver, and community education • Professional development	SLPSt	219	Academic	Hand Search
Sharma et al. [57]	2012	AUS	JA	QN	XS	SLP	Written assessment and performance assessment by SLPs	• Administered immediately after training, post-15th patient, post-30th patient • 35-item test with an 80% accuracy requirement • Interview with assessors after every 5 patients to discuss competence • Rating is given based on this interview and observation of assistant in sessions to indicate perceived competence or not competent • Assistant completes VAS with a score 0 = “not comfortable performing the tasks using the assessment” to a score of 10 = “very comfortable performing tasks during the assessment”	• Competency of CSE • Theoretical knowledge of dysphagia • Competency in treatment	• Theoretical knowledge of dysphagia • Role of SLP in dysphagia assessment and management • Feeding/positioning for swallow assessment	Allied Health Assistant	1	Telerehab	PubMed & World Cat
Urban & Hazelwood [55]	2019	USA	PA	N/A	NAS	SLP	DCVT	• Completed by self or supervisor • No detailed information was provided	• Use/know- ledge of instrumenta- tion • Competency of CSE • Competency in treatment	• CSE • Dysphagia treatment • Instrumental assessment (VFSS, FEES, HRM) • Specialization and professional development	• SLPSt • SLP Clinical Fellows • SLP	N/A	Medical SLP	World Cat
Yoshida et al. [58]	2020	Japan	JA	QN	DES	NUR	FEES education program	• Trainees’ autonomy was scored based on four major FEES skills by the head of the program • 6-point Likert scale with responses 1–6 (1 = “clear failure” to 6 = “excellent/equal to the level of trainer) 0) physician took over 1) physician provided guidance 2) physician sometimes provided guidance 3) no guidance needed • Overall evaluation score ranged from 1 = “clear failure” to 6 = “excellent/equal to the level of trainer”	• Use/know- ledge of instrumenta- tion	• Basic knowledge of FEES • FEES Procedures • Cause, prevention, and care for five adverse events • Clean-up methods for FEES • Knowledge of morphology and function • Appropriate selection of the next examination • Condition of bolus swallowing • Appropriate recommendations and training plans	NUR	3	Research facility	World Cat

*Country: AUS* Australia, *UK *United Kingdom, *USA* United States, *Record Type:* *JA* journal article, *PA* periodical article, *N/A* not applicable. *Data Type*: *QN* quantitative, *QL* qualitative, *MIX* mixed methods. *Study Design: DES* descriptive, *NAS* not a study, *XS* cross-sectional. *Author Discipline/Population of Interest*: *MDSt* medical student, *SLP* speech-language pathologist, *SLPSt* speech-language pathology student, *NUR* nursing, *PSYSt* psychology student. *Assessment/Skills:* *MGH* Massachusetts General Hospital, *CSE* clinical swallow evaluation, *DCVT* Dysphagia Competency Verification Tool, *FEES* fiberoptic endoscopic evaluation of swallowing, *HRM* high-resolution manometry, *OME* oral motor examination, *VAS* visual analog scale, *VFSS* videofluoroscopic swallow study. *Database:* *CINAHL* Cumulated Index in Nursing and Allied Health Literature

### Publication Year

**Fig. 2:**
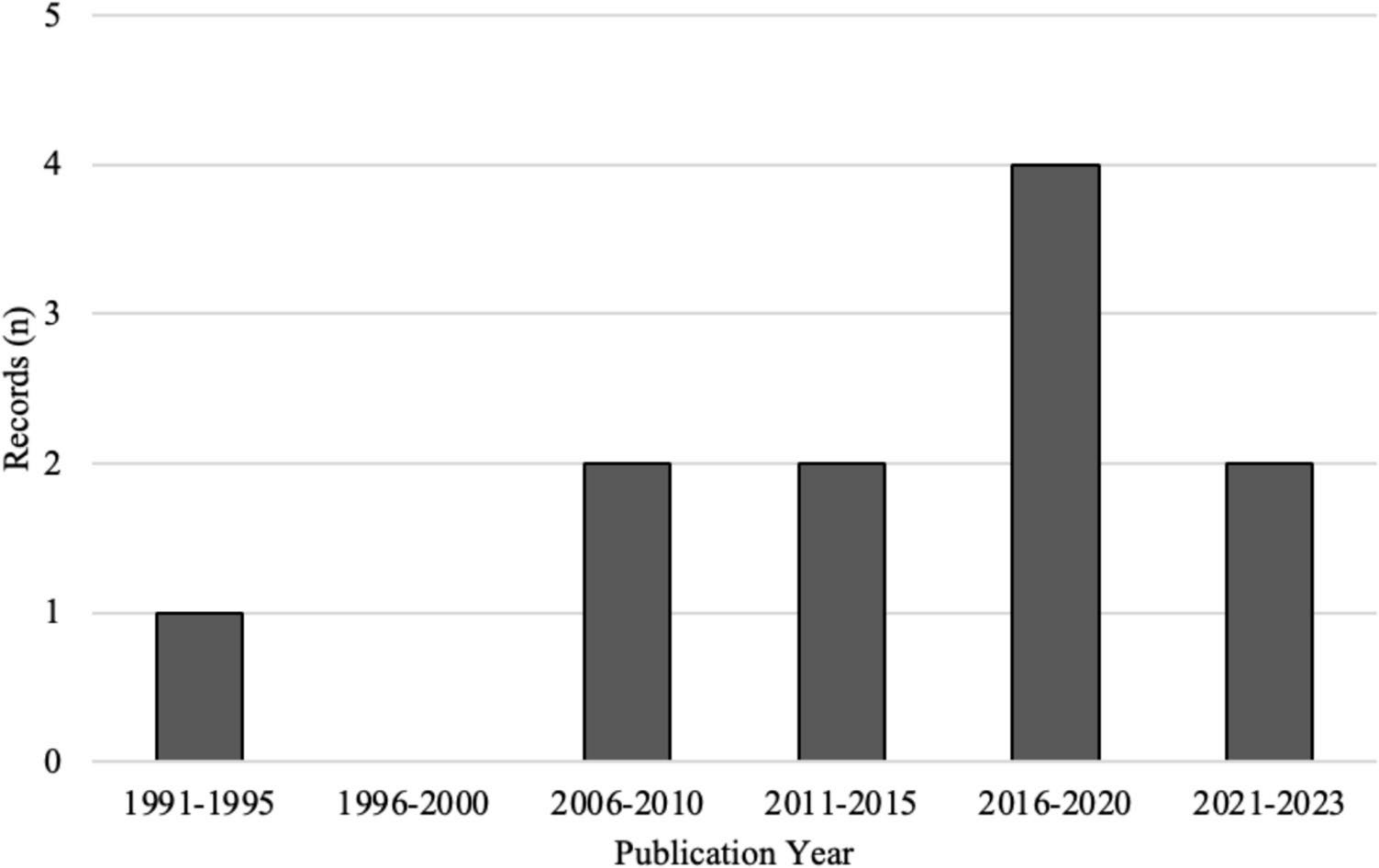
Number of records by publication year (*n* = 11)

The included records ranged from 1991 to 2022, with most (*n* = 6) published in 2016 or later [19, 32, 37, 53–55]. See Fig. 2**.**

### Publication Country

**Fig. 3:**
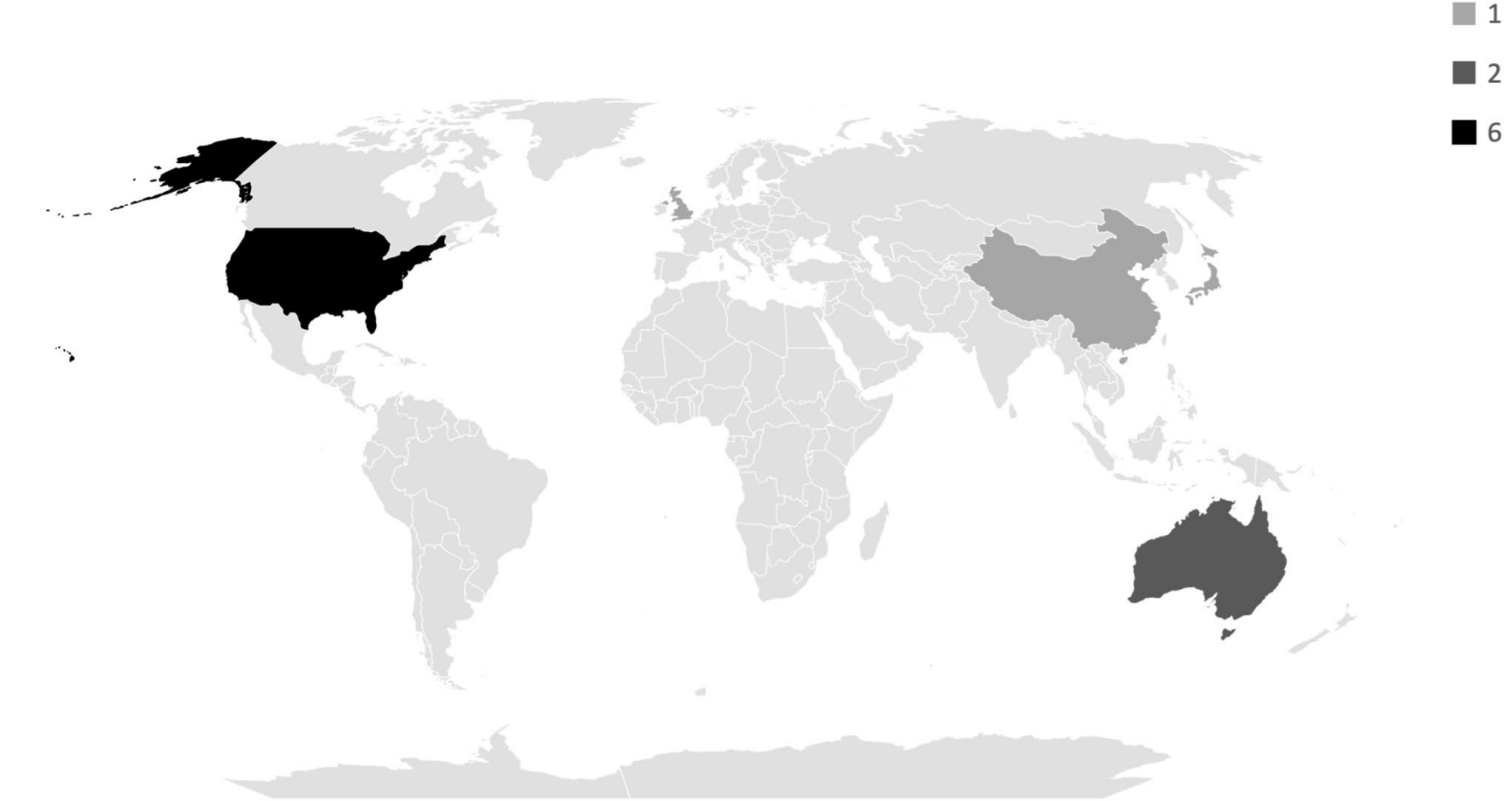
Record publication frequency by country (*n* = 11). *Map* *Citation:* Powered by Bing, Australian Bureau of Statistics, GeoNames, Microsoft, Navinfo, Open Places, OpenStreetMap, TomTom, Zenrin

More than half of the records were published by authors in the United States of America [32, 37, 52, 53, 55, 56]. However, two authors were from Australia [42, 57], one from Japan [58], one from China [19], and one from the United Kingdom [54]. Notably, no records originated from South American or African countries. See Fig. 3**.**

### Record Type

Three types of records were among the 11 included final records. The majority of the records (*n* = 9) were journal articles [19, 32, 37, 52–54, 56–58], while one record was a periodical [55], and one was a doctoral thesis [42].

### Data Type

Two records were not experimental studies [52, 55]. Seven records presented quantitative data [19, 32, 37, 53, 56–58], while the remaining two presented qualitative and quantitative data [42, 54].

### Study Type

The final 11 records included various study designs. Two of the records reported information that was not presented as a study [52, 55]. Additionally, six records were presented as cross-sectional studies [19, 32, 37, 53, 56, 57]. Two records were presented as mixed methods studies [42, 54]. One record was presented as a prospective, descriptive study [58].

### Author Discipline

Most of the records were authored by a speech-language pathologist (SLP) [37, 42, 52, 53, 55–57]. Three were authored by professionals in the nursing field [19, 32, 58], and one was authored by a medical student [54].

### Assessment Protocols

The following protocols were applied: the Dysphagia Competency Verification Tool (DCVT) [37, 55], a Massachusettes General Hospital (MGH) protocol for developing independence in the diagnosis and treatment of dysphagia with practicing clinicians (on-the-job training) [52], a competency fair [32], a medical mannequin training including didactic training and assessment [56], a video and live competency performance assessment [53], a visual analog scale (VAS) [42, 54], a questionnaire [19], a written and performance assessment [57], and a fiberoptic endoscopic evaluation of swallowing (FEES) education program [54], none of which had clear standardization criteria. Notably, all the applied assessment protocols were designed for providers of adult patients.

### Assessment Protocol Scoring Methods

The search records used multiple protocol methods to assess healthcare providers’ competence. Three records had protocols that used VAS scoring [42, 54, 57], and three other records used a Likert or ordinal scale [37, 58, 58]. Two records had protocols that used a performance assessment [52, 56]. Five of the 11 included records had protocols to be administered as a self-assessment [19, 37, 42, 54, 55]. Five records discussed protocols that could be administered by an SLP [37, 52, 53, 55, 57], and three discussed protocols that could be administered by another trained healthcare professional [32, 56, 58]. Three records had protocols that can be administered as a self-assessment *or* by a trained healthcare professional [37, 42, 55].

### Assessment Protocol Focus

The search records had protocol with multiple assessment foci. Three records discussed protocols that address providers’ understanding and theoretical knowledge of dysphagia, including signs and symptoms [19, 52, 57]. Six records described protocols that measure providers’ clinical competency when conducting clinical swallow evaluations [37, 42, 52, 53, 55, 57]. Four records assessed the providers’ knowledge or demonstration of instrumental assessments [37, 52, 55, 58]. Three records discussed study protocols that assess the competency of the providers’ use and knowledge of videofluoroscopy swallow studies (VFSS) [37, 52, 55]. Three records discussed protocols that assess providers’ use and knowledge of FEES [37, 55, 58]. Only one record described a protocol that assesses competencies in high-resolution manometry (HRM) [55]. Seven records discussed protocols that assess the healthcare providers’ competency in treating dysphagia [19, 32, 37, 42, 52, 55, 57]. Two records discussed protocols that assess providers’ competence in related clinical areas of dysphagia management, including nutrition, aphasia, and dysarthria [32, 54].

### Skills Assessed

A wide variety of skills were assessed within the final 11 records. Providers’ administration and/or understanding of swallow screenings and clinical swallow evaluations were assessed within five assessment protocols [37, 52, 53, 55, 56]. Competence of administration and/or understanding of instrumental and non-instrumental assessments were discussed in multiple records [19, 37, 42, 52, 55, 57, 58]. Three records discussed protocols that assess the providers’ knowledge and understanding of the theoretical knowledge of dysphagia [19, 52, 57]. Two records discussed protocols that assess the accuracy and timeliness of providers’ documentation skills [52, 53]. Two assessment protocols assess the providers’ ability to effectively communicate with interdisciplinary professionals, including providing relevant referrals [52, 53]. Additionally, three assessment protocols assessed the providers’ knowledge and/or ability to educate and counsel patients, caregivers, and other professionals on preventing, managing, and treating dysphagia [32, 42, 52]. Furthermore, seven assessment protocols assessed the providers’ knowledge and/or implementation of appropriate treatment techniques and programs for individual patients [19, 32, 37, 42, 52, 55, 57]. However, none of the records described the specific skills assessed regarding the providers’ knowledge or implementation of the treatment techniques and programs in depth.

### Population of Interest

**Fig. 4:**
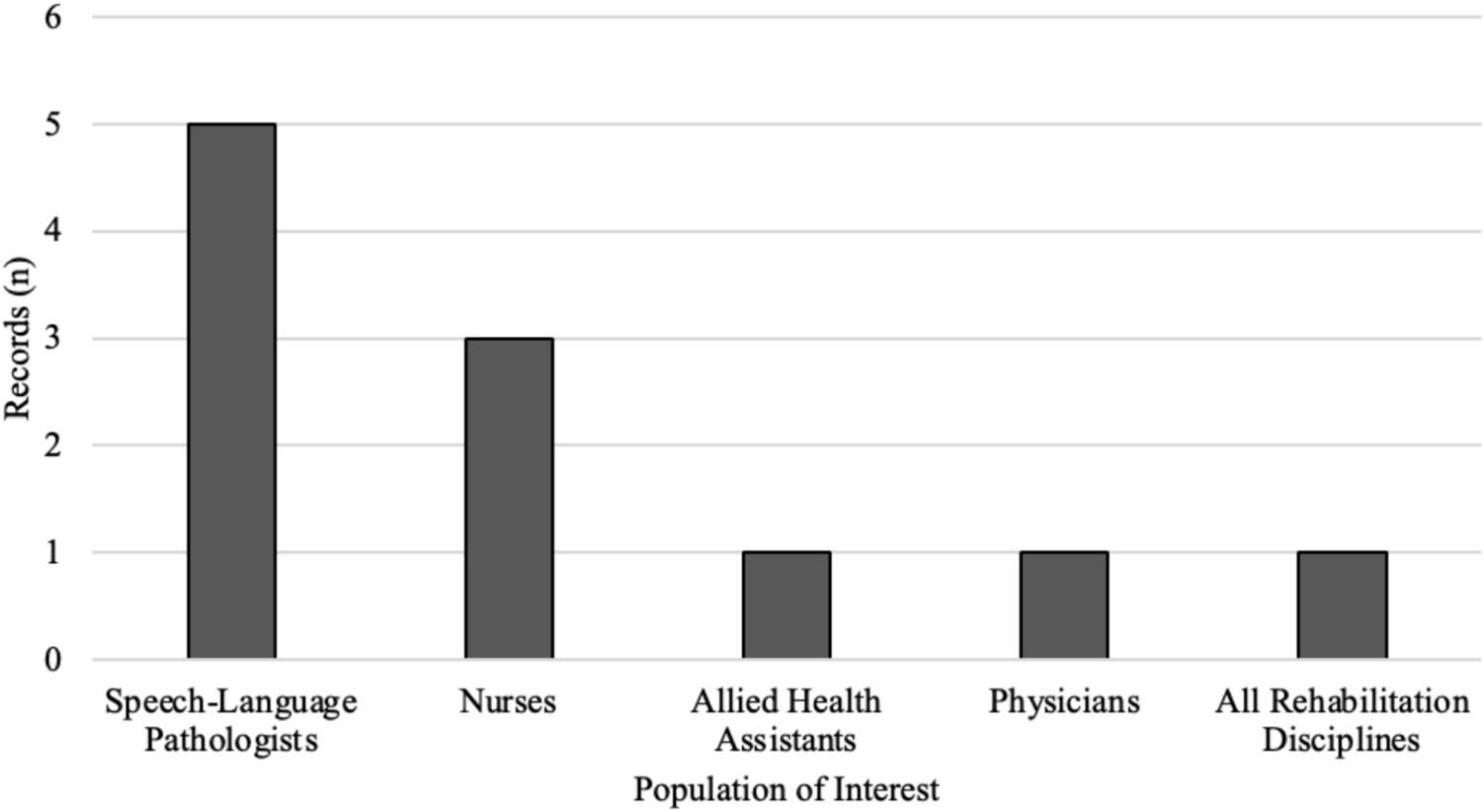
Number of records by population of interest (*n* = 11)

The specific populations of interest in the 11 final records focused on professional healthcare providers and/or their student trainees. Five records focused only on SLPs [37, 42, 52, 53, 55], three focused only on nurses [19, 56, 58], one focused on allied health assistants [57], one focused only on physicians [54], and one focused on all rehabilitation disciplines (e.g. occupational therapy, physical therapy, speech-language pathology, nursing, and other unspecified rehabilitation disciplines) [32]. See Fig. 4. Of note, six records included students of the corresponding discipline [37, 42, 52–55].

### Sample Size

Nine of the 11 final records included human subject participants [19, 32, 37, 42, 53, 54, 56–58]. Of these nine studies, the mean sample size was 160.4 participants, with a median of 72 ranging from 1 to 782.

### Participant Setting

Most studies were conducted in medical [19, 52, 55, 58] and academic [37, 42, 53, 54] settings. One study was conducted in a rehabilitation setting [32], and another used telerehabilitation [57].

### Database

Three records were retrieved exclusively from WorldCat [52, 55, 58], PubMed [19, 32, 54], or Digital Commons Network [37]. Three records were retrieved from PubMed and another database: PubMed and WorldCat [56, 57] and PubMed and CINAHL [53]. One resource was retrieved from a hand search [42].

## Discussion

Most of the final records were cross-sectional, studying SLPs, and published in peer-reviewed journal articles in the United States within the last decade. This decreased professional diversity and restricted geographic focus highlight the global need for standardized competency assessments [59]. Provider competencies included in the assessment protocols emphasized dysphagia assessment over treatment, with most protocols focusing on clinical and instrumental swallowing evaluations. However, no standardized protocol was identified for assessing dysphagia competence across healthcare providers. Students were included in over half of the study protocols, implying growing attention to pre-professional training, particularly in preparing students for dysphagia competency [60]. Despite the emphasis on competency in education, this focus does not carry over into professional careers, revealing a gap between training expectations and ongoing clinical support [35]. This lack of continuity is further reflected in the absence of clear competencies and guidelines for dysphagia management across healthcare professions, confirming that no standardized method for assessing competency exists.

The reviewed competency assessments exhibited a wide range of content, emphasizing theoretical knowledge, competency in clinical and instrumental evaluation skills, and competency in treatment techniques. We found three records providing evidence of the acquisition of theoretical knowledge [19, 52, 57]. These records emphasize the importance of understanding the pathophysiology and management of dysphagia, which is the foundation upon which further competencies are developed. Five records included the assessment of providers’ abilities to conduct clinical swallow evaluations [19, 37, 42, 55, 57]. These evaluations are integral to the early identification and management of dysphagia, ensuring that practitioners can translate theoretical knowledge into clinical practice effectively. Four records explored assessing competencies for using and understanding instrumentation in dysphagia evaluation [19, 37, 55, 57]. This focus on instrumentation highlights the critical role that technology and diagnostic tools play in accurately assessing and managing dysphagia. Six records assessed providers’ competencies regarding treatment programs and techniques [19, 37, 42, 54, 57], demonstrating the effective management of dysphagia through appropriate therapeutic interventions. Five records address participant performance and action, evaluating the participants’ skills when swallowing screenings and assessment measures are administered [32, 52, 53, 57, 58]. These records give insight into the practical application of acquired skills in real-world scenarios, reflecting providers’ transition from competence to performance and action. Two additional records extend this assessment to treatment skills during therapy sessions, affirming the providers’ ability to translate knowledge and competence into effective clinical action [52, 57].

Our findings revealed various assessment metrics employing multiple modalities to evaluate healthcare providers’ competency in dysphagia management. These measures can generally be classified into three categories: (1) broad assessments, which include self-reports or knowledge tests [19, 37, 42, 52, 54, 55, 57], (2) narrow assessments, which focus on particular assessment components or competencies, such as FEES, without evaluating the entire program [53, 54, 56, 58], and (3) unclear or unspecified assessments [32]. A prominent trend was the reliance on competency metrics that involved expert observation of specific clinical skills with a notable absence of comprehensive measures that combine both a broad scope and an operationally defined skill set. Given concerns with training variability and the prevalence of medical errors in dysphagia management [38, 61], standardized competency evaluation metrics with both self-report and expert observation of knowledge, specific skills, and global ratings would increase the accuracy and reliability when evaluating specific clinical competencies in dysphagia management across healthcare professions [43, 62]. Ideally, multiple assessment methods are needed to comprehensively evaluate a healthcare provider’s competence in all aspects of dysphagia management. No single assessment type can fully capture and measure all competency areas [43, 63]. Our review identified a notable lack of competency-based assessments for dysphagia management, which is surprising given the breadth of assessment and intervention needed. Given this, a specific methodology or metric has not been identified as the gold standard for assessing competence in dysphagia management in any field of study.

Without clear, universally applied competencies, collaboration becomes more challenging among the several dysphagia management healthcare professions, which ultimately impacts the quality, consistency, and safety of dysphagia management. There are documented inconsistencies in professional training programs, practice requirements, institutional guidelines, and professional roles during the screening and evaluation processes [30, 64, 65]. This contributes to clinical inconsistencies, including referral patterns for patients at risk for dysphagia [66], diet advancement and discrepancies in texture modifications protocols when a dysphagia specialist has yet to be consulted [67], terminology used for instrumental swallowing study reports [68], and treatment planning [61, 68]. Having a gold standard to assess competence in dysphagia management could reduce these inconsistencies and improve coordinated patient care.

### Limitations

Our research question is inherently biased as it assumes that competence in dysphagia management is being assessed among healthcare providers. However, we acknowledge that if providers are not undergoing observations or reporting self-assessment in their clinical practice of dysphagia management, no evidence for metrics to assess competence in dysphagia would have been generated in our search. Given this, if internal metrics are used within pre-/professional training facilities, these metrics will likely not be reported in peer-reviewed literature. Additionally, although our search revealed initial thesis work [42], our data corpus did not include the resulting published competency metric [69]. Lastly, this study does not represent more recent data available since the initial search and non-English sources.

### Future Studies

The findings of this scoping review indicate the need to develop a standardized metric to assess competence in dysphagia management among healthcare providers. Such a measure would have great value in standardizing what is needed to provide a high level of care to patients with dysphagia, regardless of the provider’s profession. Competency assessments should target participatory decision-making, clinical skills, core knowledge, clinical reasoning, technology use, and patient-provider relationships as assessment categories, especially when developing assessments for population-specific competencies [36, 46], such as pediatric patients.

Additionally, future research should explore the feasibility of developing and implementing competency measures that account for cultural diversity and adapt to the unique needs of various settings, including those with limited resources [17, 70]. This includes examining how cultural differences influence the perception and implementation of competencies, ensuring that measures are contextually relevant and culturally sensitive. Furthermore, research should address the challenges and practical considerations of introducing such measures in resource-constrained environments, identifying strategies to support their integration, such as training, material accessibility, and stakeholder engagement. By addressing these factors, future studies can contribute to a more inclusive and globally applicable framework for competency evaluation.

## Conclusion

This scoping review explored current literature to identify how competence in dysphagia management is assessed among healthcare providers. Existing research on this topic is limited. Though the records analyzed in this scoping review reveal varying assessments of competence in dysphagia management among healthcare providers, no standardized protocol was identified. Additionally, there is no single standardized methodology used to collect data regarding competence variables among the records reviewed, nor is there evidence for the validity and reliability of these records. The findings of this scoping review suggest that a standardized metric to assess competence in dysphagia among healthcare providers is needed to increase consistency among healthcare providers’ training towards competence in dysphagia management. Future standardized protocols should consider interdisciplinary assessment, allowing healthcare providers to improve education opportunities to meet appropriate competence standards, leading to improved patient care and health outcomes.

## Appendix A

## Data Availability

Data extracted for analysis during this study are included in this published article as required by the Preferred Reporting Items for Systematic reviews and Meta-Analyses Extension for Scoping Reviews (PRISMA-ScR) statement.

## Search Strategy

**Table:**

Databases	Search strategy (Completed February 4, 2023)		*n*
PubMed	#1	("deglutition"[MeSH Terms] OR "Deglutition Disorders"[MeSH Terms:noexp] OR "deglut*"[Text Word] OR "swallow*"[Text Word] OR "dysphag*"[Text Word])	482
	#2	("Professional Competence"[MeSH Terms] OR "Clinical Competence"[MeSH Terms] OR "health knowledge, attitudes, practice"[MeSH Terms] OR "Knowledge"[MeSH Terms])	7718
	#3	#1 AND #2	259
CINAHL	#1	MH competenc*	10,310
	#2	MH (dysphag* OR deglut* OR swallow*)	12,752
	#3	#1 AND #2	23
Digital Commons Network – Medicine and Health Sciences	#1	(deglutition OR dysphagia)	1802
	#2	(competence OR competency)	55,403
	#3	#1 AND #2	390
WorldCat	#1	(Dysphagia OR deglutition) Filters: [Book: thesis, dissertation] AND [Article, Chapter: article and downloadable article]	85,173
	#2	(competency OR competence) Filters: [Book: thesis, dissertation] AND [Article, Chapter: article and downloadable article]	1,861,877
	#3	#1 AND #2	605

## References

[1] Kababie-Ameo R, Gutiérrez-Salmeán G. Dysphagia as a predictor of malnutrition risk in older adults: a brief review of literature. Proc Sci Res Univ Anáhuac Multidiscip J Healthc. 2021;1:37–44. 10.36105/psrua.2021v1n1.05

[2] Allen B, Saunders J. Malnutrition and undernutrition: causes, consequences, assessment and management. Medicine (Baltimore). 2023;51(7):461–8. 10.1016/j.mpmed.2023.04.004

[3] Reber E, Gomes F, Dähn IA, Vasiloglou MF, Stanga Z. Management of dehydration in patients suffering swallowing difficulties. J Clin Med. 2019;8(11):1923. 10.3390/jcm8111923 31717441 10.3390/jcm8111923 PMC6912295

[4] Viñas P, Bolívar-Prados M, Tomsen N, Costa A, Marin S, Clavé P. Prevalence of dehydration among adult patients with oropharyngeal dysphagia: A systematic and scoping review. Clin Nutr ESPEN. 2023;54:566. 10.3390/nu14122497

[5] Bosch G, Comas M, Domingo L, Guillen-Sola A, Duarte E, Castells X, Sala M. Dysphagia in hospitalized patients: Prevalence, related factors and impact on aspiration pneumonia and mortality. Eur J Clin Invest. 2023;53(4):e13930. 10.1111/eci.13930 36477740 10.1111/eci.13930

[6] Vesey S. Dysphagia and quality of life. Br J Community Nurs. 2013;18:S14–9. 10.12968/bjcn.2013.18.Sup5.S14 10.12968/bjcn.2013.18.sup5.s14 23752289

[7] Hazelwood RJ, Armeson KE, Hill EG, Bonilha HS, Martin-Harris B. Relating physiologic swallowing impairment, functional swallowing ability, and swallow-specific quality of life. Dysphagia. 2023;38(4):1106–16. 10.1007/s00455-022-10532-3 36229718 10.1007/s00455-022-10532-3 PMC10097835

[8] Nativ-Zeltzer N, Nachalon Y, Kaufman MW, Seeni IC, Bastea S, Aulakh SS, Makkiyah S, Wilson MD, Evangelista L, Kuhn MA, Sahin M. Predictors of aspiration pneumonia and mortality in patients with dysphagia. The Laryngoscope. 2022;132:1172–6. 10.1002/lary.29770 34313344 10.1002/lary.29770

[9] Marin MF, Bilodeau-Houle A, Morand-Beaulieu S, Brouillard A, Herringa RJ, Milad MR. Vicarious conditioned fear acquisition and extinction in child–parent dyads. Sci Rep. 2020;10(1):17130. 10.1038/s41598-020-74170-1 33051522 10.1038/s41598-020-74170-1 PMC7555483

[10] Bohilha HS, Simpson AN, Ellis C, Mauldin P, Martin-Harris B, Simpson K. The one-year attributable cost of post-stroke dysphagia. Dysphagia. 2014;29:545–52. 10.1007/s00455-014-9543-8 24948438 10.1007/s00455-014-9543-8 PMC4179977

[11] Attrill S, White S, Murray J, Hammond S, Doeltgen S. Impact of oropharyngeal dysphagia on healthcare cost and length of stay in hospital: a systematic review. BMC Health Serv Res. 2018;18:594. 30068326 10.1186/s12913-018-3376-3 PMC6090960

[12] Shune SE, Namasivayam -MacDonald Ashwini. Dysphagia-related caregiver burden: moving beyond the physiological impairment. Perspect ASHA Spec Interest Groups. 2020;5:1282–9.

[13] Rangira D, Najeeb H, Shune SE, Namasivayam-MacDonald Ashwini. Understanding burden in caregivers of adults with dysphagia: a systematic review. Am J Speech Lang Pathol. 2022;31:486–501. 34962832 10.1044/2021_AJSLP-21-00249

[14] Pizzorni N. Social and Psychologic Impact of Dysphagia. In: Ekberg O, editor. Dysphagia Diagn Treat [Internet]. Cham: Springer International Publishing; 2019 [cited 2024 Sep 9]. p. 873–86. Available from: 10.1007/174_2017_132

[15] Labeit B, Kremer A, Muhle P, Claus I, Warnecke T, Dziewas R, et al. Costs of post-stroke dysphagia during acute hospitalization from a health-insurance perspective. Eur Stroke J. 2023;8:361–9. 37021194 10.1177/23969873221147740 PMC10069210

[16] Smith R, Bryant L, Hemsley B. The true cost of dysphagia on quality of life: The views of adults with swallowing disability. Int J Lang Commun Disord. 2023;58:451–66. 36479787 10.1111/1460-6984.12804 PMC10946621

[17] Solomon M, Coutts KA. The use of diet modifications and third-party disability in adult dysphagia: The unforeseen burden of caregivers in an economically developing country. S Afr J Commun Disord. 2020;67:1–8. 10.4102/sajcd.v67i1.777 PMC7736647 33314953

[18] Chadwick DD, Jolliffe J, Goldbart J. Carer knowledge of dysphagia management strategies. Int J Lang Commun Disord. 2002;37:345–57. 12201982 10.1080/13682820210137196

[19] Luo C-R, Wei J-Y, Zhang X-M. A multicenter cross-sectional survey of the knowledge, attitudes, and practices of geriatric nurses regarding dysphagia care. Ann Palliat Med. 2022;11:165–125. 10.21037/apm-21-3672 35144394

[20] Nielsen AH, Kaldan G, Nielsen BH, Kristensen GJ, Shiv L, Egerod I. Intensive care professionals’ perspectives on dysphagia management: A focus group study. Aust Crit Care. 2023;36:528–35. 35610091 10.1016/j.aucc.2022.04.004

[21] Chen J, Ye W, Zheng X, Wu W, Chen Y, Chen Y. Predictors of medical staff’s knowledge, attitudes and behavior of dysphagia assessment: A cross-sectional study. Rose S, editor. PLOS ONE. 2024;19:e0301770. 10.1371/journal.pone.0301770 PMC10997058 38578772

[22] Zuercher P, Moret CS, Dziewas R, Schefold JC. Dysphagia in the intensive care unit: epidemiology, mechanisms, and clinical management. Crit Care. 2019;23:103. 30922363 10.1186/s13054-019-2400-2 PMC6438038

[23] Caesar LG, Kitila M. Speech-language pathologists’ perceptions of their preparation and confidence for providing dysphagia services. Perspect ASHA Spec Interest Groups. 2020;5:1666–82.

[24] Wilkinson JM, Codipilly DC, Wilfahrt RP. Dysphagia: evaluation and collaborative management. Am Fam Physician. 2021;103:97–106. 33448766

[25] Wieseke A, Bantz D, Siktberg L, Dillard N. Assessment and early diagnosis of dysphagia. Geriatr Nur (Lond). 2008;29:376–83. 10.1016/j.gerinurse.2007.12.001 19064135

[26] Miles A, McFarlane M, Kainth P, Parmar P. Interdisciplinary management of dysphagia. Nurs Resid Care. 2014;16:561–7.

[27] Kelly H, Cronin M, Hynes H, Duxbury S, Twomey O. Learning to swallow together: Medical and speech and language therapy student interprofessional learning about dysphagia. Adv Commun Swallowing. 2021;24:21–32.

[28] McGinnis CM, Homan K, Solomon M, Taylor J, Staebell K, Erger D, et al. Dysphagia: interprofessional management, impact, and patient-centered care. Nutr Clin Pract. 2019;34:80–95. 30580461 10.1002/ncp.10239

[29] Gilles I, Filliettaz SS, Berchtold P, Peytremann-Bridevaux I. Financial barriers decrease benefits of interprofessional collaboration within integrated care programs: results of a nationwide survey. Int J Integr Care. 2020;20:10. 32256254 10.5334/ijic.4649 PMC7101009

[30] Reeves S, Pelone F, Harrison R, Goldman J, Zwarenstein M. Interprofessional collaboration to improve professional practice and healthcare outcomes. Cochrane Effective Practice and Organisation of Care Group, editor. Cochrane Database Syst Rev [Internet]. 2017 [cited 2025 Mar 25];2018. Available from: http://doi.wiley.com/10.1002/14651858.CD000072.pub3 10.1002/14651858.CD000072.pub3 PMC6481564 28639262

[31] Sánchez-Sánchez E, Avellaneda-López Y, García-Marín E, Ramírez-Vargas G, Díaz-Jimenez J, Ordonez FJ. Knowledge and practice of health professionals in the management of dysphagia. Int J Environ Res Public Health. 2021;18:2139. 33671732 10.3390/ijerph18042139 PMC7926391

[32] Browner CM, Bessire GD. Developing and implementing transdisciplinary rehabilitation competencies. SCI Nurs Publ Am Assoc Spinal Cord Inj Nurses. 2004;21:198–205. 15794419

[33] Graner D, Pressman H, Wagner LCB. Maintaining SLPs as the preferred providers of dysphagia services: a call to action. Perspect Swallowing Swallowing Disord Dysphagia. 2010;19:121–5.

[34] Carnaby GD, Harenberg L. What is “Usual Care” in dysphagia rehabilitation: a survey of USA dysphagia practice patterns. Dysphagia. 2013;28:567–74. 23670700 10.1007/s00455-013-9467-8

[35] Vose AK, Kesneck S, Sunday K, Plowman E, Humbert I. A survey of clinician decision making when identifying swallowing impairments and determining treatment. J Speech Lang Hear Res. 2018;61:2735–56. 30458527 10.1044/2018_JSLHR-S-17-0212 PMC7242916

[36] Epstein RM, Hundert EM. Defining and assessing professional competence. JAMA. 2002;287:226–35. 11779266 10.1001/jama.287.2.226

[37] Hazelwood RJ, Bouldin ED, Burford IR, Steffen EA. Speech-Language Pathology Graduate Student Clinicians’ Self-Perceived Competency in Dysphagia Management. Teach Learn Commun Sci Disord [Internet]. 2022 [cited 2024 Sep 9];6. Available from: https://eric.ed.gov/?id=EJ1365347

[38] Lee C, Namasivayam-MacDonald A, Wadhwaniya Z, McLaren J, Affoo R. A Survey of Early-Career Speech-Language Pathologists: Determining Perceived Readiness for Clinical Management of Adults with Dysphagia After Completing Graduate School. Teach Learn Commun Sci Disord [Internet]. 2024;8. Available from: https://ir.library.illinoisstate.edu/tlcsd/vol8/iss3/12.

[39] Moraes DP, de Andrade CRF. Quality indicators for integrated care of dysphagia in hospital settings. J Soc Bras Fonoaudiol. 2011;23:89–94. 21552739 10.1590/s2179-64912011000100018

[40] Speyer R, Cordier R, Sutt A-L, Remijn L, Heijnen BJ, Balaguer M, et al. Behavioural interventions in people with oropharyngeal dysphagia: a systematic review and meta-analysis of randomised clinical trials. J Clin Med. 2022;11:685. 35160137 10.3390/jcm11030685 PMC8836405

[41] ASHA. 2020 Certification Standards in Speech-Language Pathology [Internet]. Am. Speech-Lang.-Hear. Assoc. American Speech-Language-Hearing Association; [cited 2024 Sep 6]. Available from: https://www.asha.org/certification/2020-slp-certification-standards/

[42] McAllister SM. Competency based assessment of speech pathology students’ performance in the workplace. 2005 [cited 2024 Sep 6]; Available from: https://ses.library.usyd.edu.au/handle/2123/1130

[43] Bashook PG. Best practices for assessing competence and performance of the behavioral Health workforce. Adm Policy Ment Health Ment Health Serv Res. 2005;32:563–92. 10.1007/s10488-005-3265-z 16082797

[44] Hager P, Gonczi A, Athanasou J. General issues about assessment of competence. Assess Eval High Educ. 1994;19:3–16.

[45] Miller G. The assessment of clinical skills/competence/performance. Acad Med [Internet]. 1990 [cited 2024 Sep 6];65. Available from: https://journals.lww.com/academicmedicine/abstract/1990/09000/the_assessment_of_clinical.45.aspx. 10.1097/00001888-199009000-00045 2400509

[46] Lichtenberg JW, Portnoy SM, Bebeau MJ, Leigh IW, Nelson PD, Rubin NJ, et al. Challenges to the assessment of competence and competencies. Prof Psychol Res Pract. 2007;38:474–8.

[47] Tricco AC, Lillie E, Zarin W, O’Brien KK, Colquhoun H, Levac D, et al. PRISMA extension for scoping reviews (PRISMA-ScR): checklist and explanation. Ann Intern Med. 2018;169:467–73. 30178033 10.7326/M18-0850

[48] Zotero | Your personal research assistant [Internet]. [cited 2024 Sep 6]. Available from: https://www.zotero.org/download/.

[49] Covidence - Better systematic review management [Internet]. [cited 2024 Sep 6]. Available from: https://www.covidence.org/.

[50] Wolford G, Brannick SF, Strother S, Wolford L. Clinical Education Outcomes and Research Directions in Speech-Language Pathology: A Scoping Review. Teach Learn Commun Sci Disord [Internet]. 2021;5. Available from: https://ir.library.illinoisstate.edu/tlcsd/vol5/iss2/3.

[51] Moghabghab R, Tong A, Hallaran A, Anderson J. The difference between competency and competence: a regulatory perspective. J Nurs Regul. 2018;9:54–9.

[52] Arsenault J, Atwood J. Development of a competency-based training model for dysphagia management in a medical setting. Semin Speech Lang. 1991;12:236–45.

[53] Hoepner JK, Hemmerich AL. Using formative video competencies and summative in-person competencies to examine preparedness for entry-level professional practice. Semin Speech Lang. 2020;41:310–24. 32698227 10.1055/s-0040-1713782

[54] Kennedy G, Rea JN, Rea IM. Prompting medical students to self–assess their learning needs during the ageing and health module: a mixed methods study. Med Educ Online [Internet]. 2019 [cited 2024 Sep 6];24. Available from: https://www.tandfonline.com/doi/full/10.1080/10872981.2019.1579558 10.1080/10872981.2019.1579558 PMC6508056 31046637

[55] Urban M, Hazelwood RJ. Are You Ready to Manage Dysphagia? ASHA Lead [Internet]. 2019 Jan 19 [cited 2024 Sep 6]; Available from: https://leader.pubs.asha.org/doi/10.1044/leader.OTP.24072019.38

[56] Freeland TR, Pathak S, Garrett RR, Anderson JA, Daniels SK. Using medical mannequins to train nurses in stroke swallowing screening. Dysphagia. 2016;31:104–10. 26519043 10.1007/s00455-015-9666-6

[57] Sharma S, Ward E, Burns C, Theodoros D, Russell T. Training the allied health assistant for the telerehabilitation assessment of dysphagia. Sage J [Internet]. 2012 [cited 2024 Sep 6];18. Available from: https://journals.sagepub.com/doi/10.1258/jtt.2012.111202 10.1258/jtt.2012.111202 22790011

[58] Yoshida M, Kagaya H, Kamakura Y, Miura Y, Saitoh E, Okawa Y, et al. Safety and the effectiveness of a new education program for nurses to assess swallowing function using fiberoptic endoscopic evaluation of swallowing (FEES). Jpn J Nurs Sci [Internet]. 2020 [cited 2024 Sep 6];17. Available from: https://onlinelibrary.wiley.com/doi/abs/10.1111/jjns.12313 10.1111/jjns.12313 31883217

[59] Jogerst K, Callender B, Adams V, Evert J, Fields E, Hall T, et al. Identifying interprofessional global health competencies for 21st-century health professionals. Ann Glob Health. 2015;81:239. 26088089 10.1016/j.aogh.2015.03.006

[60] Affoo R, Bruner J, Dietsch A, Nellenbach C, Jones T, Lehman M. The Impact of Active Learning in a Speech-Language Pathology Swallowing and Dysphagia Course. Teach Learn Commun Sci Disord [Internet]. 2020;4. Available from: https://ir.library.illinoisstate.edu/tlcsd/vol4/iss2/4.

[61] Plowman EK, Humbert IA. Elucidating inconsistencies in dysphagia diagnostics: Redefining normal. Int J Speech Lang Pathol. 2018;20(3):310–7. 10.1080/17549507.2018.1461931 29724130 10.1080/17549507.2018.1461931

[62] Nandamudi S, McKnight A, Baird K. Dysphagia interprofessional collaborative practice and team-based learning in graduate curriculum for students in healthcare disciplines: A pilot study. J Interprofessional Educ Pract. 2023;30: 100588. 10.1016/j.xjep.2022.100588

[63] Miller E, Colquhoun H. The importance and value of reporting guidance for scoping reviews : A rehabilitation science example. Aust J Adv Nurs 2020;37(4)53–8. 10.37464/2020.374.148.

[64] Mathers–Schmidt BA, Kurlinski M. Dysphagia evaluation practices: inconsistencies in clinical assessment and instrumental examination decision-making. Dysphagia. 2003;18:114–25. 10.1007/s00455-002-0094-z 12825905 10.1007/s00455-002-0094-z

[65] Reddacliff C, Hemsley B, Smith R, Dalton S, Jones S, Fitzpatrick A, Given F, Kelly J, Lawson X, Darcy S, Debono D. Examining the content and outcomes of training in dysphagia and mealtime management: a systematic review informing co-design of new training. Am J Speech Lang Pathol. 2022;31(3):1535–52. 10.1044/2022_AJSLP-21-00231 35377733 10.1044/2022_AJSLP-21-00231

[66] Lorinczi K, Denheyer V, Pickard A, Lee A, Mager DR. Referral criteria for assessment and treatment: in an ambulatory dysphagia clinic. Can J Diet Pract Res. 2012;73(4):189–94. 10.1186/s12910-023-00885-1 23217446 10.3148/73.4.2012.189

[67] O’Keeffe ST, Leslie P, Lazenby-Paterson T, McCurtin A, Collins L, Murray A, Smith A, Mulkerrin S. SPARC (Swallow Perspectives, Advocacy and Research Collective). Informed or misinformed consent and use of modified texture diets in dysphagia. BMC Med Ethics. 2023;24(1):7. 10.1186/s12910-023-00885-1 36750907 10.1186/s12910-023-00885-1 PMC9903443

[68] Rameau A, Katz P, Andreadis K, Drenis S, Joseph IT, Tran A, Han G, Sarhadi KS, Kaufman M, Belafsky P. Clarifying inaccurate terminology: the important difference between dysphagia and swallowing dysfunction. Foregut J Am Foregut Soc. 2022;2(1):11–7. 10.1177/26345161211072761

[69] McAllister, S., Lincoln, M., Ferguson, A. & McAllister, L. COMPASS®: Competency assessment in speech pathology Melbournetechnical manual. 2nd ed. Melbourne (AU). Melbourne: Speech Pathology Australia; 2013. Available at: https://www.speechpathologyaustralia.org.au/Common/Uploaded%20files/Smart%20Suite/Smart%20Library/5eb81d01-bd01-47b8-b636-bb05d9d443b3/COMPASS%20-%20Technical%20Manual.pdf

[70] Mustaffa Kamal R, Ward EC, Cornwell P, Sharma S. Provision of dysphagia services in a developing nation: Infrastructural challenges. Int J Speech Lang Pathol. 2015;17(6):594–604. 10.3109/17549507.2015.1026276 25874970 10.3109/17549507.2015.1026276

